# Lessons from the EU AI Act

**DOI:** 10.1016/j.patter.2025.101183

**Published:** 2025-02-14

**Authors:** Alejandra Alvarado

**Affiliations:** Scientific editor, *Patterns*, Cell Press

## Main text

Last year, the Artificial Intelligence Act came into force in the European Union (EU), establishing a framework for the development, commercialization, and application of AI within the EU. The goal is to promote human-centric AI while making sure fundamental rights of citizens are protected. The act presents a classification of AI systems based on their potential risks, outlines obligations for AI commercial providers, and establishes an “AI office” to provide guidance and manage AI-related risks. Importantly, the act establishes a requirement for transparency, mentioning options like metadata and watermarking for identifying AI-generated content. In the United States, on the other hand, the executive order "Safe, Secure, and Trustworthy Development and use of AI" issued last year, which outlined the country’s approach to advancing and regulating AI, led to the publication of a report by the National Institute of Standards and Technology (NIST) on tools and methods to help manage the risk of AI. Although this executive order was recently rescinded, its intentions aligned with the AI Act's focus on transparency and accountability. What do these developments mean for researchers?

We believe the requirements in the AI Act won’t change the landscape regarding the detection of covert AI content. Scientists have tried to leverage intrinsic features of text, like word distribution or probability curvatures, but as detection tools improve, so do the tools to evade detection (see Mitchell et al., 2023, *International Conference on Machine Learning*, PMLR). Simply, the continued evolution of AI technology has and will continue to make many detection methods futile, as AI tools become increasingly more sophisticated in their ability to generate human-like text. As mentioned earlier by Christianson, 2024, this has become a race where no one is going to win. Indeed, recent studies have found that the accuracy of AI detectors tends to be below 80%, and some tools struggle to identify paraphrased or modified AI-generated text (Weber-Wulff et al., 2023, *Int. J. Educ. Integr.*). Additionally, these detectors often misclassify human-written work as AI generated. Watermarking and other tools to identify AI content are unlikely to fundamentally change this arms race since the mark-up could also be removed.

But AI users could still learn from the requirements in the Act and the overview presented by the NIST. Arguably researchers should be investigating tools, like those mentioned in the AI Act, to mark-up AI-generated content. We encourage our authors to consider *providing metadata or using watermarking even for research grade tools and models*. We believe our best choice is *being transparent about AI use and ensuring text and in general all digital assets are traceable*. Ultimately, traceability is important for reproducibility, and both transparency and reproducibility are central for science.

As we mentioned earlier, we are convinced that our best alternative is to combine approaches, that is to promote human-AI collaboration and transparent disclosure of AI usage. By utilizing AI to augment and enhance the writing process, researchers and academic writers can benefit from the efficiency and creativity that these tools offer while maintaining the intellectual rigor and authenticity that are essential to academic discourse.

